# Meta-analysis as an emerging trend to scrutinize the effectiveness of L2 pragmatics instruction

**DOI:** 10.3389/fpsyg.2022.1016661

**Published:** 2022-11-22

**Authors:** Farzaneh Shakki

**Affiliations:** Department of English Language and Literature, Faculty of Humanities and Social Sciences, Golestan University, Gorgan, Iran

**Keywords:** meta-analysis, review, pragmatics, instruction, research

## Abstract

How efficient is instruction in pragmatics? We have attempted to answer this question through meta-analyses. Considering the plethora of studies conducted in L2 pragmatics instruction, it is still challenging for researchers to keep up with the literature, so aggregating the findings across multiple studies and comparing their results systematically in various dimensions can be pivotal to deciding whether this kind of research is effective or not. This review paper delineates the previous meta-analyses and reviews conducted in the field of instructed second language pragmatics in EFL/ESL context to explore the importance of conducting meta-analyses and to recommend some suggestions and pedagogical implications for further studies.

## Introduction

For centuries, researchers have known that the inevitable existence of sampling error in single studies may lead to variation in the results of the studies. This variation seems to be a weakness in methodology and that is why all studies recommend more research in that field of research (Hunter and Schmidt, 2004). Taking this problem into account, the only solution to sampling error is the cumulation of findings from different studies. Meta-analysis as a solution to this problem was posited by Glass (1976), and “it refers to a quantitative review of the research on the effect of the certain treatment on a response variable” (Li, 2010, p. 312). According to Hall et al. (1994), the aim of meta-analysis is to “summarize and add new knowledge” (p. 24, 25). Furthermore, a meta-analysis arranges a clear explanation of the findings of every study using a numerical index of effect size and it mixes these results of the previous single studies to come to the conclusion across rudimentary research. Since its commencement, meta-analysis has become widespread in psychology, education, and biomedical sciences as a way of using statistics to combine the results of primary studies to check the effectiveness of a variable (Hedges, 1992).

Due to its advantages over the previous approaches used (the vote-counting method, the “cumulation of *p*-values” method, and the narrative method), meta-analysis has been considered an emerging method of research (Borenstein et al., 2021). Meta-analytic studies started long ago, though the first draft had not been released until the 1930s. There were two different kinds of meta-analysis at that time, one of them dealt with the combination of estimates, and the other concerned the combination of hypotheses (Crombie and Davies, 2009). Due to the fact that this innovative trend has not been adequately addressed in language learning and it has been mostly used in medical issues, the benefits of this research method is worth mentioning. First, meta-analysis is done rigorously; consequently, the danger of bias can be overcome and this contributes to detecting significant and notable results of studies (Derakhshan and Shakki, 2021). Second, unlike individual studies, meta-analysis can provide a lucid picture through the meta-regression technique in which instead of too few participants, aggregated data is used. Last but not least, the transparency of meta-analysis can be another advantage that helps readers to identify the rationality of the decisions taken through the process of finding the effect sizes (Crombie and Davies, 2009).

Considering its advantages, meta-analysis is becoming prolific; as a result, applying it in language studies has been recommended (Oswald and Plonsky, 2010). One of the essential factors in language learning is instruction that was analyzed for its effectiveness numberless in the last decades. Following the seminal research of Norris and Ortega (2000), which was on the effectiveness of instruction, the supremacy of meta-analysis compared with other research methods has been recognized and identified. Since then, the amalgamation of studies has been carried out so far through meta-analyses on different subjects such as corrective feedback (Russell and Spada, 2006; Yousefi and Nassaji, 2019), the effectiveness of negotiated interaction in SLA (Mackey and Goo, 2007), construct validity of language aptitude (Li, 2016), the process writing approach (Graham et al., 2012), and form-focused instruction (Kang et al., 2019). Reviewing the meta-analyses that were carried out, it was found that the instruction of pragmatics has received scant attention, and it was only analyzed regarding one of its features namely the speech act by covering many moderators in an Iranian context (Shakki et al., 2020, 2021, 2023). This paper reviewed previous studies to pave the way for future studies in instructed second language pragmatics.

Pragmatics was described by Morris (1938) as “the study of the relation of signs to interpreters” (p. 6), and it has been considered very fertile ground for research. Crystal (1985) also spotted that the process of language use by which coding and decoding by interlocuters happens has a vital role in pragmatics research. Similarly, Mey (2001) states that “Pragmatics studies the use of language in human communication as determined by the conditions of society” (p. 6). He elucidates that “the use of language needs to be negotiated between the users of language themselves in their social and communicative relations and linguistic interactions” (Mey, 1993, p. 315).

As a vital and indispensable component of language competence, pragmatics has increasingly come to the fore. Learners of Second Language (L2) experience significant difficulty in learning pragmatics, mostly due to the complexity of pragmatics, which involves more than just focus-on-form(s). The vista of pragmatic competence has been revamped from one-to-one association within form, function, and context of use, and it is now believed that the form–function–context correspondences are not fixed among individuals (Taguchi, 2015). In addition, adult learners, unlike children whose pragmatic and linguistic competence grows at the same time, face many challenges in the process of pragmatic development because of their first-language interferences.

Reviewing the previous findings on pragmatics illustrated slow pragmatic development in a realistic setting (Taguchi, 2019). The majority of scholars believe that pragmatic features, like other language skills such as grammar and vocabulary, should be included in classroom pedagogy (Shakki et al., 2016). Researchers examined the effectiveness of different instructional methods, such as input- and output-based instruction, explicit and implicit teaching, meta-pragmatic discussion, and teaching within the zone of proximal development (ZPD) (Vygotsky, 1987; Alcón-Soler and Martínez-Flor, 2005; Kasper and Roever, 2005; Martínez-Flor and Alcón-Soler, 2005; Rose, 2005; Cohen, 2008; Takahashi, 2010a,b; Taguchi, 2011, 2018, 2019; Birjandi and Derakhshan, 2014; Derakhshan and Eslami, 2015; Culpeper et al., 2018; Derakhshan and Arabmofrad, 2018; Blyth and Sykes, 2020; Derakhshan and Eslami Rasekh, 2020; Derakhshan and Shakki, 2020; Irshad and Bukhari, 2020; Malmir and Derakhshan, 2020; Tajeddin and Alemi, 2020; Derakhshan and Cohen, 2021; Derakhshan and Malmir, 2021; Derakhshan et al., 2021; Hernández, 2021). The findings from previous studies show the positive effects of instruction and its superiority.

Moreover, some scholars carried out systematic reviews and meta-analyses on pragmatic instruction (Jeon and Kaya, 2006; Plonsky and Zhuang, 2019; Yousefi and Nassaji, 2019; Shakki et al., 2021) and claimed that teaching pragmatics is believed to be more effective for L2 pragmatic features. Considering the substantial prominence of Interlanguage Pragmatics (ILP) in learning and teaching, and in order to check the effectiveness of pragmatic instruction, meta-analyses are recommended to bridge the gap and to check whether the amalgamation of studies that have been carried out so far are in harmony. Through meta-analysis in this area, some variables that can predict the effectiveness of pragmatic instruction, especially the speech acts of request, apology, and refusal, can be found. Finally, the purpose of the present study is to summarize the conducted review and meta-analyses studies in the area of L2 pragmatics instruction to date to establish its importance for future research.

## Review of the literature

### Instruction of pragmatics

Interlocuters need to know each other's intentions and the appropriate ways of using English in each new situation, so there is a need for instruction in order to have successful communication (Sánchez-Hernández and Alcón-Soler, 2020). Individuals must be instructed about how to interpret the meaning in the context and how to have better negotiations. Instructed second language acquisition as a subcategory of second language acquisition occurs as a result of combining the teaching of some materials in the classroom with independent study to help students learn how to use the target language in everyday life. Second language instruction is a context in which teachers try to guide and facilitate the process of learning by assisting learners to achieve a better proficiency level.

Kasper and Rose (1999, 2002) found that pragmatics was amenable to being taught, and that instructed groups often outperformed non-instructed groups. This represents what was elucidated by Ellis (2008) about instruction, the more input, the more opportunities to produce output and better language learning. In order to avoid any misunderstanding, instruction as a pivotal factor can be assumed and has its roots in the noticing hypothesis. Regarding the instruction of pragmatics, which was considered to be effective during past decades (Alcón-Soler and Martínez-Flor, 2005; Taguchi, 2011, 2015, 2019; Culpeper et al., 2018; Blyth and Sykes, 2020; Derakhshan and Shakki, 2020; Derakhshan et al., 2020), Rose and Kasper (2001) stated that all pragmatic features, namely hedges, speech acts, and address markers, are amenable to instruction, and this claim was analyzed by many scholars (Bagherkazemi, 2018; Derakhshan and Arabmofrad, 2018; Kaivanpanah and Langari, 2020; Malmir, 2020) and is supported by the results of this review paper. Recent years have witnessed a plethora of studies on the effect of instruction on different pragmatic features (Jeon and Kaya, 2006; Plonsky and Zhuang, 2019; Shakki et al., 2021; Matsumura, 2022), and it pinpoints the advantages of meta-analysis over other methods of research to assess this effectiveness.

### Empirical studies

After Kasper's plenary talk, which inspired the investigation into the effectiveness of instruction, several reviews and meta-analyses have been carried out on instruction and instructed pragmatics. Norris and Ortega (2000), for instance, used 49 studies that were published between 1980 and 1998. They planned to ascertain the effectiveness of instruction through focus on forms and focus on form studies. They concluded that the explicit groups (focused L2 instruction) performed better than the other counterpart. This study was among the first meta-analyses done on the effectiveness of second language instruction.

Moreover, Jeon and Kaya (2006) carried out the first meta-analysis in instructed pragmatics approximately 14 years ago by using only 13 studies prior to 2003. Their results demonstrated that explicit and direct instruction provides a dramatic difference over no instruction group. Considering the effect sizes, Cohen *d* was 0.70 for the experimental vs. control group in explicit, though it was 0.44 for the implicit group. For pretest, posttest in the explicit group *d* was 1.9, whereas the implicit instruction was *d* = 1.01. Since the number of studies utilized in their study was limited, further meta-analyses were proposed.

Almost 5 years later, in another paper, a review article was written by Taguchi (2015) on the development of pragmatic instruction over the past three decades. She employed 58 studies in her paper and she also found that instruction is more beneficial than non-instruction. Taguchi believes that explicit instruction is more beneficial than implicit, though through some activities and consciousness-raising, it can be promoted.

A year later, and in a similar way, Badjadi (2016) used 24 studies to determine the impacts of second language pragmatics' instruction related to comprehension and production outcome measures. The effect size change was from small to large and it corroborated the effectiveness of instruction. In addition, Plonsky and Zhuang (2019) distributed two research questions to see the effectiveness of instruction and to check the moderator variables that may predict this efficiency. They utilized 50 studies and their results also are in accordance with the previous meta-analyses in which the importance of explicit instruction was accentuated over implicit instruction. They reported *d* = 1.52, which is quite a large effect size for the overall effectiveness of instruction. They also believed that the instruction of pragmatics by providing some opportunities is more effective than instruction without opportunities. Furthermore, they stated that longer instruction and role play produce larger effect sizes than their counterparts.

In another study, Yousefi and Nassaji (2019) analyzed 39 studies from 2006 to 2016 on the effects of corrective feedback and instruction on L2 pragmatics. The findings of their study showed a larger effect size for computer-assisted instruction in comparison with face-to-face intervention. Taking comprehension into consideration, it produced a larger effect size than the production of second language pragmatic. Moreover, the intermediate learners and longer treatments both generated larger effect sizes than other language proficiency levels and shorter interventions.

Furthermore, Shakki et al. (2020) also reviewed 54 studies carried out on the instruction of pragmatics from 2000 to 2020 in an Iranian context, and they reported that request was the most frequently instructed speech act in the last two decades which has been used in 29 studies. They also found Multiple-Choice Discourse Completion Test (MCDCT) to be the dominant data collection method in Iranian studies, and the most recurrent treatment type used in Iran was found to be explicit/implicit vs. control.

By the same token, Derakhshan and Shakki (2021) conducted a meta-analysis on the effectiveness of the instruction of request in the Iranian context. Using special inclusion/exclusion criteria, they selected 17 primary studies and the analysis corroborated the effectiveness of instruction in L2 pragmatics in an Iranian context. They found that gender and treatment type can be considered moderator variables for this effectiveness. The results showed a larger effect size for males (*g* = 3.09) than females (*g* = 1.10), and explicit (*g* = 1.53) rather than implicit (*g* = 1.20). The overall effectiveness of instruction was found to be *g* = 1.48, which is positive and large for teaching request.

Recently, the latest meta-analysis by Shakki et al. (2021) was conducted on the effectiveness of instruction for the speech act of apology. Among 31 studies, they used 12 primary studies to check their research questions. The medium effect size was found in this study for the overall effectiveness of apology instruction, and the research design (*g* = 2.39) was the variable that moderates this productivity and it is assumed to be the predictor for this efficiency. However, the treatment types also generated medium and large effect sizes for the instruction of apology in pragmatics.

A critical look at the studies conducted revealed that they have used a limited number of papers based on their inclusion and exclusion criteria, and most of them have selected some of the variables to check their moderating roles. It seems that among all studies, the effectiveness of instruction with the variety of effect sizes ranging from 0.07 to 1.52 has been approved, and generally explicit instruction generates larger effect size than implicit instruction. A limitation of this study is that it has only focused on the papers in which pragmatics instruction was analyzed to see whether meta-analysis is effective enough in this field to be used for further research. Other review papers could be carried out on other aspects of pragmatics or other research topics to pave the way for further studies.

## Conclusion and suggestions for further research

Meta-analyses presented a summative description of the primary studies that have been done so far, and the findings revealed the effectiveness of the variables and identified the factors that moderate this efficiency. It could resolve the difficulty of determining whether the research conducted was successful or not, and could be taken into account as innovative and emerging trends for future publications. Meta-analyses aim to generate quantitative estimates of primary studies by combing data to identify the effect sizes. Since the larger sample size brings greater reliability, meta-analyses are also very reliable and precise in conclusion by increasing the generalizability. Considering the empirical studies, previous meta-analyses can help researchers to perceive the magnitude of the effect sizes better, and they could lead to identifying vital trends and conclusions that could influence policymakers' decisions and future research. Among the implications and strengths of meta-analyses, model testing, high statistical power, and moderator analyses can be carried out, which make them more replicable and systematic than qualitative and traditional reviews.

Although doing meta-analyses has been found to be fruitful, one of the pervasive problems in using them is missing data or unpublished papers that are not available to researchers, so it is recommended that researchers contact people who are experts in the field to see whether they have any unpublished papers, or whether they know of any conferences occurring. Furthermore, researchers are advised to avoid including even one bad study in meta-analysis, because instead of solving the problem of variance, the study could ruin the entire sample (Field and Gillett, 2010). Thus, defining clear and precise inclusion and exclusion criteria is also advisable.

The number of meta-analyses investigated pragmatic instruction which was a concern in this study is scant; therefore, more meta-analyses need to be done to have pivotal pedagogical implications for second language pragmatics and future research. While searching in previous meta-analyses for speech acts, we were surprised to find that there are some speech acts, such as threats, condolences, congratulations, and challenges, that have received scant attention, so future studies could be conducted in these neglected areas. The only meta-analyses carried out on the effectiveness of speech acts (Derakhshan and Shakki, 2021; Shakki et al., 2021) have examined requests and apologies. Researchers could also check other treatment types, comprehension, and production of pragmatics, and could also take into account other factors such as the gender, age, and proficiency level of the learners to lead to better outcomes and to check their effectiveness.

In addition to speech acts, which are a vital feature of pragmatics, other variables, such as implicatures and routines, could also be a new research area for future studies. This paper may also be useful for researchers whose areas of interest are meta-analysis and pragmatics. They could conduct meta-analysis to check the moderator variables that are helpful predictors in teaching pragmatics, and could also work on data collection methods other than WDCT and MDCT, such as role play, to assess whether they are more reliable for the instruction of pragmatics. Another suggestion would be to focus future meta-analysis on different levels of proficiency.

## Author contributions

The author confirms being the sole contributor of this work and has approved it for publication.

## Conflict of interest

The author declares that the research was conducted in the absence of any commercial or financial relationships that could be construed as a potential conflict of interest.

## Publisher's note

All claims expressed in this article are solely those of the authors and do not necessarily represent those of their affiliated organizations, or those of the publisher, the editors and the reviewers. Any product that may be evaluated in this article, or claim that may be made by its manufacturer, is not guaranteed or endorsed by the publisher.
